# *Isaria cicadae* conidia possess antiproliferative and inducing apoptosis properties in gynaecological carcinoma cells

**DOI:** 10.1080/21501203.2017.1386243

**Published:** 2017-10-11

**Authors:** Yan-fang Sun, Yang Sun, Zhi-an Wang, Rui-lian Han, Hong-fei Lu, Jia-lei Zhang, Hong-tao Liu, Shi-xian Wang, Pan Wang, Lu-lu Dian, Zong-suo Liang

**Affiliations:** a Laboratory of Plant Secondary Metabolism and Regulation of Zhejiang Province, College of Life Sciences, Zhejiang Sci-Tech University, Hangzhou, China; b College of Resource and Civil Engineering, Northeastern University, Shenyang, China; c Zhejiang Research Institute of Traditional Chinese Medicine, Hangzhou, China; d School of Civil Engineering and Architecture, Zhejiang Sci-Tech University, Hangzhou, China

**Keywords:** *Isaria cicadae*, *Isaria cicadae* broken conidia powder (ICBCP), high performance liquid chromatography-electrospray/quadrupole time of flight tandem mass spectrometry technology (UPLC-ESI-Q-TOF-MS), gynaecological carcinoma cells, mitochondrial apoptosis pathway

## Abstract

*Isaria cicadae* is an entomogenous fungus that has been used as a traditional Chinese medicinal materials to treat different diseases, including cancer. However, *Isaria cicadae* conidia for inhibitory activity against breast cancer cells growth are still not systematically studied. The present aim was to elucidate the phytochemical composition of *Isaria cicadae* conidia and to explore relevant anti-cancer potential in gynaecological carcinoma MCF-7 and Hela cells. *Isaria cicadae* conidia were identified by UPLC-ESI-Q-TOF-MS: high performance liquid chromatography-electrospray/quadrupole time of flight tandem mass spectrometry technology. Eight main compounds were identified which are nucleosides, cordycepic acid, cordycepin, beauvericin and myriocin by MS fragmentation ions. The nuclear morphology indicated the typical characteristics of apoptosis by Hoechst staining. Annexin V/PI staining revealed that the number of apoptotic cells was increased by *Isaria cicadae* conidia treatment. Furthermore, *Isaria cicadae* conidia also induced the caspase-mediated mitochondrial apoptosis pathway. The findings suggest that the full-scale active ingredients highlight the significance of *Isaria cicadae* conidia as potential anti-cancer agent in China.

## Introduction

*Isaria cicadae*, which belongs to the Clavicipitaceous family and genus *Isaria*, is an entomogenous fungi that parasitises on nymphs of cicada, has been used as a tonic food and herbal medicine for dietary therapy, and the morphological structure of *Isaria cicadae* is mainly composed of conidia, mycelium and coremium (Weng et al. 2002; Wang et al. 2017). In addition, *Isaria cicadae* conidia are commonly known as cicadas “seed”, the formation process is as follows: the larva of cicada is infected and parasitised by *Paecilomyces* before emergence; the fungus absorbs the polypide nutrient and turns into a mycelium; the mycelium transforms from a nutritive into a sexual stage; it then gradually branches and blooms from the top, forming the “*Isaria cicadae* conidia” which is essential for reproductive functions (Wei et al. 2016).

Pharmacological studies have showed that *Isaria cicadae* possess renoprotective, neuroprotective effects and anti-cancer activities (Chyau et al. 2014; Zhu et al. 2014; Olatunji et al. 2016). However, the main chemical compositions and anti-cancer mechanisms of *Isaria cicadae* broken conidia powder (ICBCP) were still not very clear. The wild *Isaria cicadae* is more rare and precious, which limits its extensive application. *Isaria cicadae* is a variable due to the infection of different kinds of fungi that is very similar in appearance. However, differences in chemical compositions can affect the biological activities of *Isaria cicadae* conidia. The present study mainly focused on the active ingredients analysis of *Isaria cicadae* conidia cultivated in China. Furthermore, the antiproliferative potentials of *Isaria cicadae* conidia against the human breast cancer cells were investigated for the first time.

## Materials and methods

### Experimental materials

Wild *Isaria cicadae* was collected from the bamboo forest Tianmu Mountain Natural Reserve in Lin’an, Zhejiang (119°23'.47“~119°28'27“E, 30°18'30“~30°24'55“N) in June 2016, and identified by Prof. Hongxin Zhao of Fungus Taxonomy. Voucher specimens (No. PP20160628) were deposited at the Laboratory of Plant Secondary Metabolism and Regulation of Zhejiang Province, China.

Human breast cancer cells MCF-7 and human cervical cancer cells Hela were obtained from the Chinese Academy of Sciences, Shanghai Institutes for Cell Resource Center. The cell lines were maintained on Dulbecco´s modified Eagle high glucose medium (Gibco, USA), supplemented with 10% foetal bovine serum (Hyclone, USA), 100 U/mL penicillin and 100 U/mL streptomycin. The cells were cultured in an incubator with 5% CO_2_ and 95% humidity; the experiments were performed with cells in the logarithmic growth phase.

### Extraction and analysis of Isaria cicadae conidia

*Isaria cicadae* conidia were placed in a mortar, ground with liquid nitrogen and dried at 40°C for further analysis. To analyse and identify the chemical constituents from the water extract of ICBCP by UPLC-ESI-Q-TOF-MS (Waters Co., USA), the analysis was performed on an ACQUITY UPLC^TM^ HSS T3 reverse phase column (2.1 mm × 100 mm, 1.8 μm). The mobile phase consisted of acetonitrile (A) and 0.1% aqueous formic acid (B) with the gradient conditions as follows: 0 ~ 2.50 min, 1%~11% A; 2.50 ~ 6.00 min, 11%~21% A; 6.00 ~ 8.00 min, 21%~40% A; 8.00 ~ 8.50 min, 40%~60% A; 8.50 ~ 9.50 min, 60%~99% A; 9.50 ~ 11.25 min, 99% A; 11.25 ~ 11.50 min, 99%~1% A. The flow rate was 0.40 mL/min and injection volume was 10 µL. Quadrupole-time of flight-mass spectrometry was applied for the qualitative analysis under positive and negative ion modes and ESI ion source with full scan MS mode (50 ~ 1000 m/z).

### Cell viability assay

3-(4,5-dimethyl-thiazol-2yl)-2,5-diphenyl tetrazolium bromide (MTT) assay was employed to evaluate the effect of ICBCP on the viability of human lung carcinoma MCF-7 and Hela cells (Sun and Wink 2014). A known value of 1.0 × 10^4^ cells were plated in 96-well plates and treated with different concentrations of ICBCP for 24 h. A known value of 5.0 mg/mL MTT (20 µL) was added to each well and cells were incubated for another 4 h at 37°C. The reaction was terminated by addition of 150 µL dimethylsulfoxide (DMSO) and optical density at 570 nm was determined on a microplate reader (BioTek). About 50% of inhibitory concentration (IC_50_) was calculated from growth-inhibitory curves of cells by SigmaPlot 12.5 software.

#### Cell nuclear morphology observed by hoechst33342 fluorescence staining

MCF-7 and Hela (2.5 × 10^5^ cells/well) were seeded into six-well plates and incubated to 90% confluence. Subsequently, the cells were treated with different concentrations (0, 40, 80 and 160 µg/mL) of ICBCP for 24 h, and then indirectly incubated with Hoechst 33,342 (20 µg/mL) for 30 min in the dark. The dyed fluorescent cells were observed under a fluorescence microscope (Olympus, Tokyo, Japan).

### Quantitative analysis of apoptosis by flow cytometry

Apoptosis was monitored using the common Annexin V-FITC/PI detection kit (BD, Biosciences Pharmingen) as described by the manufacture’s instruction (Sun et al. 2013). MCF-7 and Hela cells in six-well plates were treated with different concentrations (0, 40, 80 and 160 µg/mL) of ICBCP for 24h, cells were collected, washed twice with Phosphate buffered saline (PBS), gently resuspended in Annexin V-binding buffer and incubated with Annexin V-FITC and PI in dark for 10 min. The fluorescent intensity of surface exposure of phosphatidylserine (PS) by apoptotic cells was measured by flow cytometry with a Coulter Cytomics FC500 through quadrant statistics for necrotic and apoptotic cell populations. Data were analysed using BD Accuri^TM^ C6 software (BD Biosciences, San Jose, CA).

### Analysis of cell cycle distribution

MCF-7 and Hela cells (2.5 × 10^5^ cells/well) in exponential growth were seeded in six-well plates and treated with different concentrations (0, 40, 80 and 160 µg/mL) of ICBCP for 24 h. After incubation, the cells were collected, centrifuged and fixed with ice-cold 70% ethanol for 4 h, then incubated with 20 µg/mL RNase A and 10 µg/mL propidium iodide (PI) for 30 min in the dark. The stained cells of cell cycle phase distribution and hypodiploid DNA were detected by flow cytometer (BD, FACSAria）at 488 nm and analysed with FlowJo software (Verity Software, Topsham, ME).

### Cell protein extraction and immunoblotting

Immunoblotting was performed as described previously (Li et al. 2015). After MCF-7 and Hela cells were treated with different concentrations of ICBCP (0, 40, 80 and 160 µg/mL) for 48 h. They were then resuspended in cold PBS after removal of the nutrient solution, and then centrifuged at 1500 rpm for 5 min and lysed in RIPA lysis buffer with freshly added 100 mM PMSF by ice bath for about 30 min; afterwards, the protein supernatant was centrifuged at 12,000 rpm for 10 min at 4^°^C. The protein supernatant was quantified using a BSA standard curve. The proteins were denatured with loading buffer at 100^°^C. A known value of 40 μg protein was separated by using 10–12% SDS-PAGE and transferred to PVDF membranes (Millipore Corporation, USA). The membranes were blocked with 5% skim milk (BD Biosciences, Franklin), which dissolved in pH-adjusted TBST at room temperature for 2 h. The membranes were incubated with diluted primary antibodies in skim milk at 4^°^C with gentle shaking, overnight, included Caspase-3 (1:1000), Caspase-8 (1:1000), Caspase-9 (1:1000), PARP (1:1000), Bcl-2 and Bax, β-actin was considered as the internal control. The membranes were incubated with HRP-linked anti-rabbit and anti-mouse IgG secondary antibodies diluted by TBST at room temperature for 2 h. The protein bands were visualised with Ultra ECL by supersensitive chemiluminescence imaging (Tanon Co., China).

## Results and discussion

### Active ingredients of ICBCP by UPLC-ESI-Q-TOF-MS analysis

A rapid and full-scale UPLC-ESI-Q-TOF-MS method was employed to elucidate chemical profiles of ICBCP. As shown in Table 1, compound 1 only showed negative ions of 181.1086[M-H]^−^ and 163.0992[M-H-H_2_O]^−^, and the element composition was C_6_H_12_O_6,_ and identified as cordycepic acid. Compound 4 showed [M + H]^+^, [M-H]^−^ ions at m/z 402.3118, 400.2919 and identical fragmentations at m/z 384.2959[M + H-H_2_O]^+^ and 801.5169[M + M-H]^−^, which displayed analogical MS and MS^2^ patterns as those of myriocin. In addition, the main fragment ions of compound 5 were analysed by MS/MS screening which observed at m/z 268.1437[M + H]^+,^ 249.0793[M-OH]^−^ and characteristic fragment ion at m/z 136.1097[M + H-2-deoxy-D-ribose]^+^, the calculated molecular formula was speculated to be C_10_H_13_N_5_O_4_ based on the analysis of its elemental composition and fractional isotope abundance 265.2649[M-^2^H]^−^, and after screening the target compounds in the formula database, the purity scores was 99%, this ion was then identified as adenosine. Finally, eight main compounds including guanosine, uridine, adenosine, N^6^-(2-hydroxyethyl) adenosine, cordycepin, cordycepic acid, myriocin and beauvericin from the water extract of ICBCP were successfully identified by comparison with the element compositions analysis, retention times, MS fragmentation ions and the reference standards.

**Table 1.** Qualitative analysis of chemical constituents in ICBCP.

		Relative molecular weight					
N0.	*t*_R_/min	Measure value	Theoretical value	Characteristic fragment	Molecular formula	Chemical compound	Fragment ions of belonging
1	9.19 (+)	402.3118	401.2780	402, 384	C_21_H_39_NO_6_	Myriocin	402[M + H]^+^; 384[M + H-H_2_0]^+^
	9.19 (^−^)	400.2919		400, 801			400[M-H]^−^; 801[2M-H]^−^
2	10.60 (+)	784.3804	783.9490	784, 885	C_45_H_57_N_3_O_9_	Beauvericin	784[M + H]^+^; 782 [M-H]^−^
	10.58 (^−^)	782.3666		339, 782			885[M + 2-methylbutanamide+H]^+^;339[D-α-Hydroxyisovaleryl-L-N-methyl-Phe+PhH]^−^
3	0.63 (^−^)	181.1086	182.1720	163, 181	C_6_H_14_O_6_	Cordycepic acid	163[M-H-H20]^−^;181[M-H]^−^
4	2.43 (+)	312.1664	311.2940	162, 180, 312	C_12_H_17_N_5_O_5_	N_6_-(2-hydroxyethyl)-adenosine	162[M-OH-2-deoxy-D-ribose]^+^;180[M + H-2-deoxy-D-ribose]^+^;
							312[M + H]^+^
5	9.08 (+)	242.3236	244.2010	124, 242	C_9_H_12_N_2_O_6_	Uridine	124[M+CH_2_-2-deoxy-D-ribose-H]^+^; 242[M-^2^H]^+^; 243[M-H]^−^
	1.56 (^−^)	243.0976		200, 243			200[M-CHCH_2_OH-H]^−^
6	1.92 (+)	282.3168	283.2410	150, 282	C_10_H_13_N_5_O_5_	Guanosine	282[M-H]^+^; 565[2M+H]^−^;
	1.89 (^−^)	282.1140	282.3168	565			150[M + H-2-deoxy-D-ribose]^−^;
							282[M-H]^−^
7	1.95 (+)	252.1483	251.2420	119, 136	C_10_H_13_N_5_O_3_	Cordycepin	119[M^−^+2H-deoxy-D-ribose]^+^;
	10.90 (^−^)			252, 178, 265			136[M+H_2_O-2-deoxy-D-ribose]+;
							252[M + H]+; 265[M+CH_2_]^−^;
							178[M-CH_2_CHOCH_2_OH+H]^−^;
8	1.82 (+)	268.1437	267.2410	136, 268, 97,	C_10_H_13_N_5_O_4_	Adenosine	136[M + H-2-deoxy-D-ribose]^+^;
		265.2549		176			268[M + H]^+^;
	10.56(^−^)			249, 265			97[M-adenine-H-2OH]^−^;
							176[M-CH_2_OHCHOCHOH-H]^−^;
							249[M-OH]^−^; 265[M-2H]^−^

### Antiproliferative activity of ICBCP

**Figure 1.**
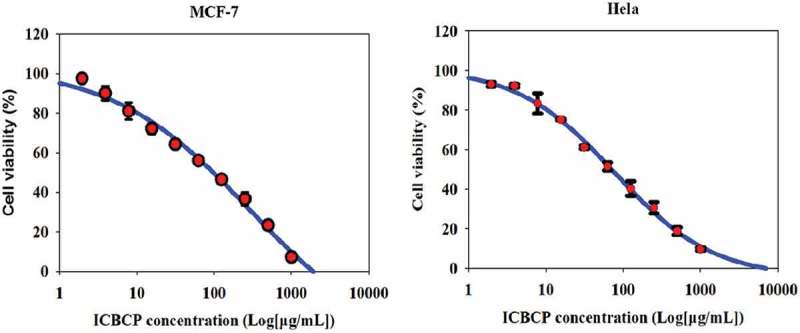
Antiproliferative effects of ICBCP on MCF-7 and Hela cells by MTT assay. The cells were incubated for 24 h with various concentrations of ICBCP, and cell viability was determined by MTT assay. Data are mean values of three experiments ± standard deviations (S.D.).

MTT assay was used to investigate cell viability of MCF-7 and Hela cells (Figure 1). ICBCP treatment resulted in a significant dose-dependent inhibition of the growth of MCF-7 and Hela cells, and the IC_50_ values were IC_50_ = 109.14 ± 4.37 and IC_50_ = 78.16 ± 5.19 µg/mL, respectively. Apparently, Hela cells were more sensitive to ICBCP than MCF-7 cells.

### Effect of ICBCP on apoptosis in MCF-7 and Hela cells

MCF-7 and Hela cells were stained with the cell permeable DNA dye by Hoechst 33342, to examine the nuclear morphological changes by fluorescent microscopy. Faintly stained, round and intact nuclei were observed in the control group, indicating typical healthy cells. However, ICBCP treated groups (40, 80 and 160 µg/mL) exhibited a gradually increasing degree of apoptosis as well as apoptotic cells with some evident morphological changes, such as improved brightness, reduced cellular volume, gradually condensing and marginalised chromatin, fragmented nucleus and formed apoptotic bodies.

**Figure 2.**
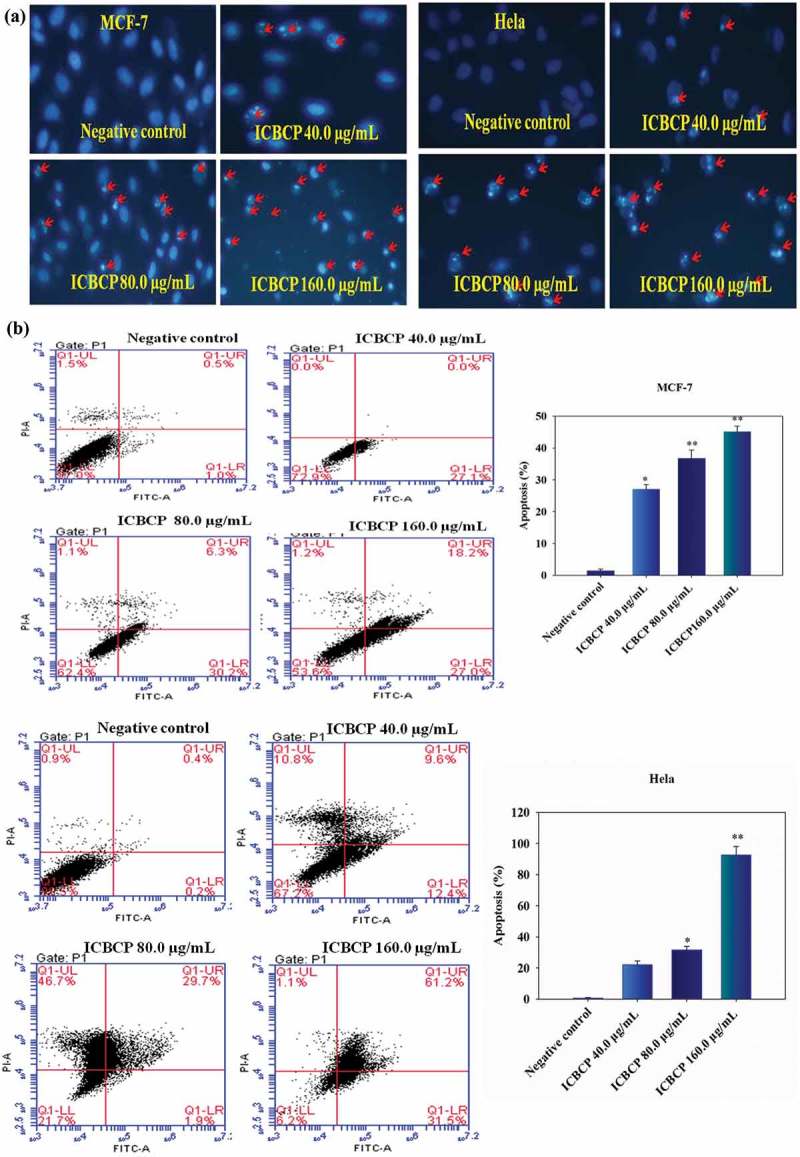
(a) Morphological observation by inverted fluorescence microscopy of MCF-7 and Hela cells stained with Hoechst 33,342 after ICBCP treatment. Photomicrographs of fluorescence staining were randomly examined with a magnification of 40. Apoptotic cells containing fragmented nuclear chromatin are indicated by red arrows. (b) ICBCP induced apoptosis in MCF-7 and Hela cells were detected from labelled Annexin V-FITC/PI by flow cytometric histogram analyses. Columns show mean values of three experiments expressed as mean ± S.D. (n = 3). Statistical analyses were performed by Student’s *t*-test (*P<0.05, **P<0.01) compared with the negative control group.

To quantify the extent of apoptosis, MCF-7 and Hela cells treated with ICBCP were stained with Annexin V-FITC/PI followed by flow cytometry analysis. The lower left quadrant contained intact cells. The lower right quadrant consisted of early apoptotic cells. The upper right quadrant contained late-apoptotic or necrotic cells. After treatment with ICBCP (Figure 2), the percentages of both early and late apoptotic cells gradually increased in a dose-dependent fashion. The percentages of MCF-7 cells undergoing apoptosis following treatment with 0, 40, 80 and 160 µg/mL ICBCP (including the early and late apoptotic cells) were 1.50 ± 0.34%, 27.10 ± 0.59%, 36.50 ± 1.20% and 45.20 ± 1.07%, respectively. Similarly, the apoptosis rates of Hela were 0.60 ± 0.19%, 22.0 ± 0.67%, 31.60 ± 1.58% and 92.70 ± 3.25%, respectively. Therefore, ICBCP promoted MCF-7 and Hela cell apoptosis in a dose-dependent manner via externalisation of PS, which is a characteristics features of cancer cell apoptosis.

### Cell cycle distribution

**Figure 3.**
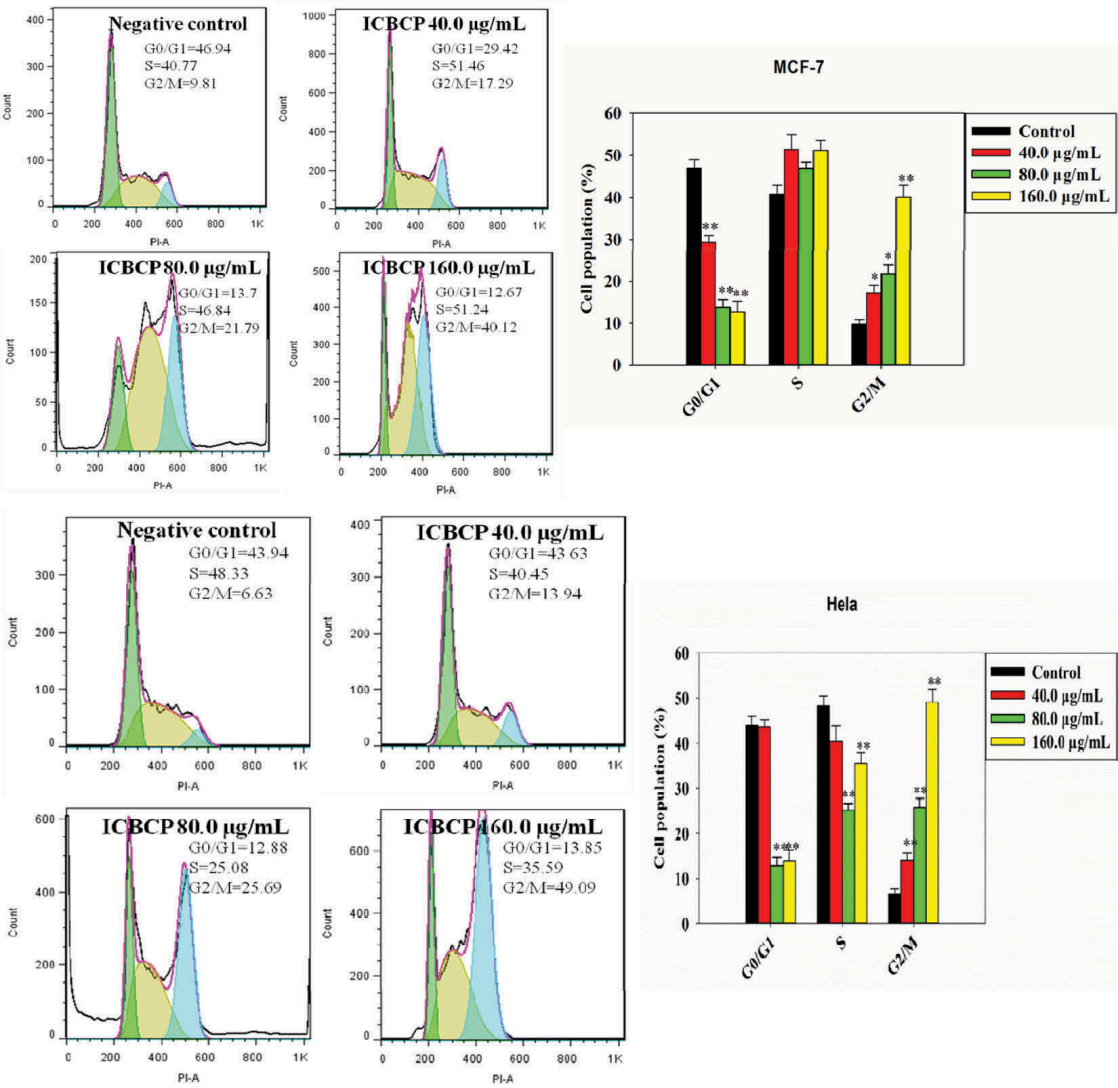
Effect of ICBCP on cell cycle in MCF-7 and Hela cells stained with PI and analysed by flow cytometer equipped with 488 nm laser. The X-axis represents the DNA content and the Y-axis represents the cell counts. The graph depicts the percentage of apoptotic cells. The data are mean ± S.D. of three independent experiments. *P<0.05 difference in the apoptotic population compared to untreated control.

MCF-7 and Hela cells were treated with ICBCP and then stained with PI as shown in Figure 3. The percentages of cells in the G0/G1 phase with a hypodiploid DNA histogram decreased from roughly 46.94% (negative control) to 12.67% for MCF-7 and from 43.94% to 13.85% for Hela cells, respectively. Subsequently, the number of cells in the G2/M phase also correspondingly elevated from 9.81% to 40.12% for MCF-7 and from 6.63% to 49.09% for Hela cells, respectively. ICBCP significantly decreased the percentages of cells in the G0/G1 phase in a dose-dependent manner and increased the percentages of cells in the G2/M phase in both cell lines. These data indicate that the ICBCP specifically induced cell cycle checkpoints and suppressed the reproduction of cancer cells associated with cycle arrest.

### Apoptotic signalling pathways

**Figure 4.**
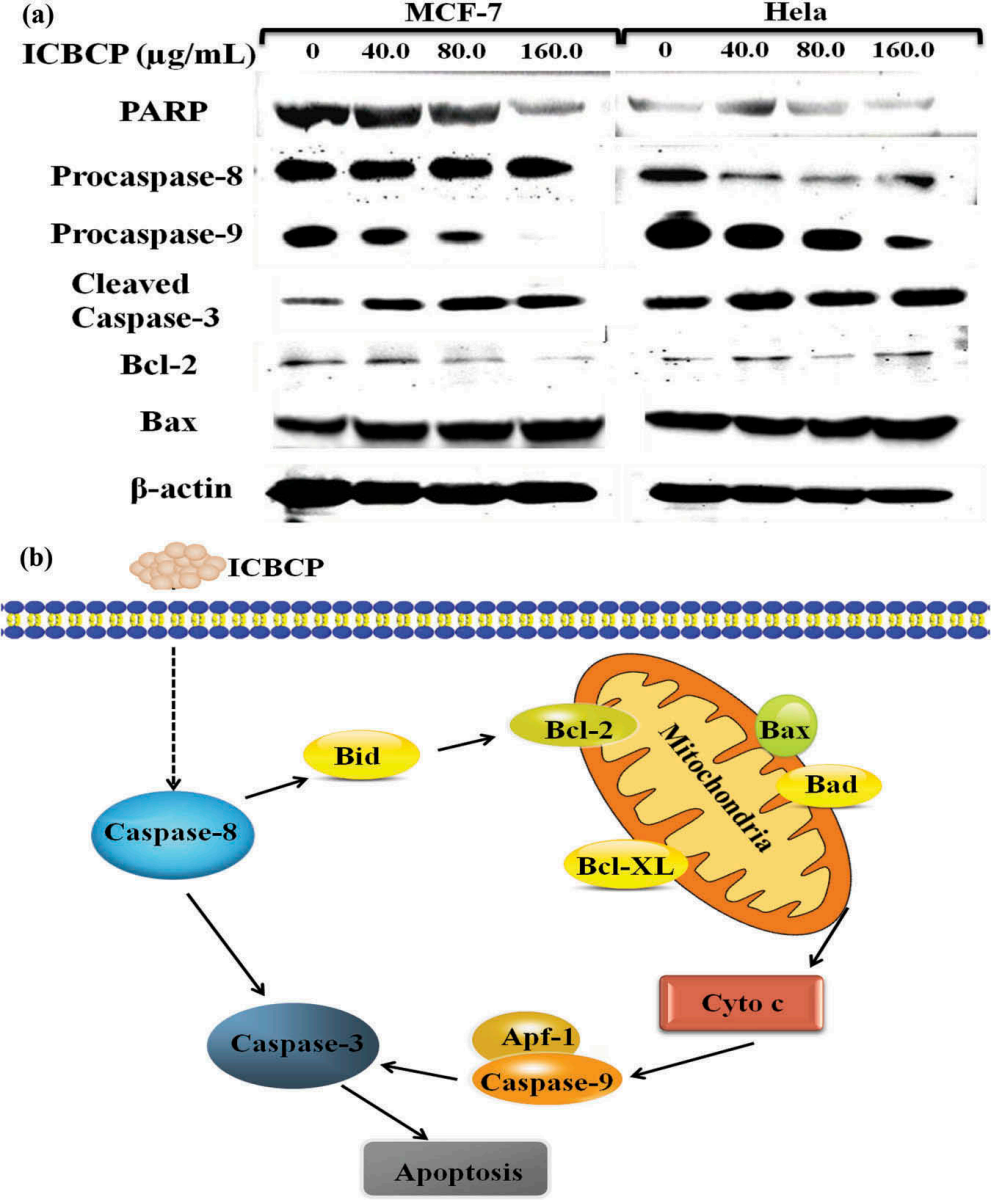
(a) Caspase induced cleavage of PARP, Caspase-3, Caspase-8, Caspase-9 and Bax and Bcl-2 expression in MCF-7 and Hela cells were determined through Western blotting after 48 h treatment. β-actin served as loading control. (b) The schematic diagram illustrates how ICBCP could regulate mitochondrial control of apoptosis pathway in MCF-7 and Hela cells.

Apoptosis is a programmed cell death, induced by a variety of extracellular stimuli and involves intracellular signalling pathways (Wyllie et al. 1984). The mitochondrial pathway is the best known intrinsic apoptosis pathway. The mitochondrion is the main site of action for members of the apoptosis-regulating protein family. Bcl-2 family proteins, including Bax and Bcl-2, regulate apoptosis by controlling mitochondrial permeability that can inhibit cell apoptosis. Bax family proteins can induce cell apoptosis via mitochondrial control of apoptosis (Hengartner 2000). MCF-7 and Hela cells were treated with increasing concentrations of ICBCP. The expression of Bcl-2, Bax, Caspase-3, Caspase-8, Caspase-9 and PARP was determined by Western blotting. Apparently, expressions of the apoptotic proteins Bax and Caspase-3 were up-regulated, whereas expressions of anti-apoptotic proteins Bcl-2, PARP, Caspase-8 and Caspase-9 were down-regulated. ICBCP could elicit apoptosis in MCF-7 and Hela cells via caspase-dependent pathway (Figure 4). The ICBCP comprises a large number of different components so that their mode of action can involve several mechanisms for inhibition of MCF-7 and Hela cells in gynaecological carcinoma.

In summary, this study provided a chemical characterisation of *Isaria cicadae* conidia, which exhibited remarkable antiproliferative properties and induced apoptosis in gynaecological cancer cells.
